# Optimization of Extraction Conditions of Carotenoids from *Dunaliella parva* by Response Surface Methodology

**DOI:** 10.3390/molecules27041444

**Published:** 2022-02-21

**Authors:** Yujia Li, Xiaojuan Huang, Lirong Luo, Changhua Shang

**Affiliations:** 1 Key Laboratory of Ecology of Rare and Endangered Species and Environmental Protection, Ministry of Education, College of Life Sciences, Guangxi Normal University, Guilin 541006, China; liyujia@stu.gxnu.edu.cn (Y.L.); zhangwenjia8@stu.gxnu.edu.cn (X.H.); gxnu202011002019@stu.gxnu.edu.cn (L.L.); 2 Guangxi Key Laboratory of Landscape Resources Conservation and Sustainable Utilization in Lijiang River Basin, Guangxi Normal University, Guilin 541006, China; 3 School of Life Sciences, Sun Yat-Sen University, Guangzhou 510275, China

**Keywords:** *Dunaliella parva*, Central Composite Design, extraction efficiency, carotenoids, optimization

## Abstract

Extraction conditions can exert a remarkable influence on extraction efficiency. The aim of this study was to improve the extraction efficiency of carotenoids from *Dunaliella parva* (*D. parva*). Dimethyl sulfoxide (DMSO) and 95% ethanol were used as the extraction solvents. The extraction time, extraction temperature and the proportions of mixed solvent were taken as influencing factors, and the experimental scheme was determined by Central Composite Design (CCD) of Design Expert 10.0.4.0 to optimize the extraction process of carotenoids from *D. parva*. The absorbance values of the extract at 665 nm, 649 nm and 480 nm were determined by a microplate spectrophotometer, and the extraction efficiency of carotenoids was calculated. Analyses of the model fitting degree, variance and interaction term 3D surface were performed by response surface analysis. The optimal extraction conditions were as follows: extraction time of 20 min, extraction temperature of 40 °C, and a mixed solvent ratio (DMSO: 95% ethanol) of 3.64:1. Under the optimal conditions, the actual extraction efficiency of carotenoids was 0.0464%, which was increased by 18.19% (the initial extraction efficiency of 0.03926%) with a lower extraction temperature (i.e., lower energy consumption) compared to the standard protocol.

## 1. Introduction

Carotenoids are important phytochemicals contributing to health benefits in the human diet. They are lipophilic pigments produced by organisms, especially plants and microorganisms [1,2,3]. Carotenoids have a terpenoid structure consisting of a long, conjugated chain. Most of the important carotenoids are tetraterpenes, which are usually condensed from eight isoprene units [4,5,6]. Carotenoids can be divided into two groups according to their structure, carbohydrate type and oxidized type. Carotenoids have important biological activities in organisms [6,7,8,9] and can play an important role in enhancing immunity, anti-oxidation and delaying aging, preventing tumor, cardiovascular and cerebrovascular diseases, and fighting cancer [10]. Carotenoids also reduce the risk of many types of cancer, metabolic syndrome, obesity, cataracts and chronic diseases such as macular degeneration [11]. Therefore, as food colorants and nutritional supplements, carotenoids are widely used in food, medicine and health care products [12,13].

Carotenoids are mainly extracted from natural organisms. *Dunaliella* are unicellular algae with higher salt tolerance that are mainly distributed in salty water and lake water. Extreme environmental conditions, such as high salt concentrations, low temperature, and the lack of nutrition, could induce the accumulation of natural carotenoids [14,15,16,17,18,19,20]. *Dunaliella* can thrive under high NaCl concentrations (0.3 to 3.0 M) [21]. Interestingly, *D. parva*, a halophilic microalga devoid of a cell wall and with high carotenoid content, has been used in the cosmetics and dietary supplements industries [14,22]. *D. parva* lacks a cell wall, which is favorable for genetic manipulation and product extraction [23,24]. Previous studies reported the tolerance of *D. parva* against various unfavorable conditions associated with nutrition, illumination, heavy metal ions and hyperosmotic shock, demonstrating the high carotenoid production of *D. parva* under the above stress conditions [25,26,27,28]. These unique traits give *D. parva* considerable advantages.

Compared with first-generation plant-based biofuels, microalgae have many potential advantages for biofuel production. Microalgae do not require agricultural land and can fix CO_2_ with higher efficiency. In addition, microalgae can be used for wastewater treatment, biogas upgrades and nutrient removal [29]. As a halophilic microalga, *D. parva* could yield lipid and abundant carotenoids and adapt to environmental stresses such as high salt concentration. 

Regarding the *Dunaliella* genus, many studies have focused on its mechanism of salt resistance. Proteomic analysis revealed the mechanism of the *Dunaliella salina* *Ds-26-16* gene by enhancing salt tolerance in *Escherichia coli* [30]. Four enzymes functioned together to keep up the glycerol requirements in order to adapt to high salt concentrations [31]. Our laboratory performed a series of in-depth studies about *D. parva*. We cloned and characterized several key genes of *D. parva* related to photosynthesis and carbohydrate and lipid metabolism [32,33,34,35,36]. We studied the changes in the transcriptome and proteome under nitrogen limitation conditions and identified the gene *DpWRI1-like* as a regulator of lipid metabolism in *D. parva* [18,19]. In addition, we cloned three carotenoid biosynthesis genes (*Psy*, *Pds* and *GGPS*) and investigated the changes of expression of three key genes [37,38]. Although our previous studies have greatly explored the lipid and carotenoid metabolism of an important microalga, *D. parva*, at the molecular level, the lack of studies about carotenoid extraction from *D. parva* has significantly limited the application and development of *D. parva*.

At present, the extraction technologies of carotenoids mainly include organic solvent extraction, green solvent extraction, microwave-assisted extraction, ultrasonic-assisted extraction and supercritical fluid extraction [39,40,41,42,43]. The organic solvent extraction method has the following advantages: the clear experiment principle, mature technology, ease of operation, and a growing public familiarity. Common organic solvents are anhydrous ethanol, ethyl acetate, 95% ethanol, n-hexane, petroleum ether, methanol solution, 2% acetic acid solution, acetone, and so on [9]. Because carotenoids belong to a class of lipid-soluble pigments, they can be dissolved by fat-soluble solvents. The objective of this study was to determine the optimal conditions for the extraction of carotenoids from *D. parva*. Based on the study of a single extraction solvent, two better extraction solvents were mixed. Through CCD of the response surface method, optimized carotenoid extraction conditions were obtained to improve the extraction efficiency of carotenoids, which laid a good foundation for the further application of carotenoids in *D. parva*.

## 2. Materials and Methods

### 2.1. Algal Species

*D. parva* (FACHB-815) was purchased from the Freshwater Algae Culture Collection at the Institute of Hydrobiology.

### 2.2. Experimental Design

In order to determine the solvent with higher extraction efficiency, the extraction efficiencies of 7 commonly used solvents (petroleum ether, ethyl acetate, 95% ethanol, n-hexane, ethanol, DMSO and acetone) were compared. These solvents were of analytical grade (Sangon Biotech). Then, two solvents with higher extraction efficiencies were mixed in fixed proportions as the mixed solvent.

In this study, three factors, including extraction time, extraction temperature and the proportions of the mixed solvent, were selected as the main influencing factors for the extraction of carotenoids from *D. parva* (Table 1). The CCD method was used to optimize the values of the influencing factors. The design was carried out using Design Expert 10.0.4.0 software. Three replicates were performed for each group.

### 2.3. Extraction of Carotenoids

The extraction of carotenoids was performed based on the former study with minor modification [44]. After culturing *D. parva*, 1 mL of culture was centrifuged at 12,000 rpm for 5 min at room temperature; then, the supernatant was discarded to obtain the precipitation. A total of 1 mL of extraction solvent was added to the precipitate and mixed by a vortex oscillator. Then, the mixture was incubated at the preset temperature (60 °C in the standard protocol) for the preset time (20 min in the standard protocol) and centrifuged at 12,000 rpm for 2 min. At last, 250 μL supernatant was added to the microplate.

### 2.4. Determination of Carotenoids Content

The absorbance values of the extract (250 μL) at wavelengths of 665 nm, 649 nm and 480 nm were measured by a BioTek Epoch 2 microplate spectrophotometer (Agilent, Santa Clara, CA, USA) in order to calculate the carotenoid content in *D. parva*. The corresponding extraction solvent was used as a blank control. The concentration of carotenoids (µg mL^−1^) and the extraction efficiency of the carotenoids (%) were calculated according to the following formulas [44].

Chla = 12.47 × A_665_ − 3.62 × A_649_ (1)

Ch1b = 25.06 × A_649_ − 6.5 × A_665_ (2)

C = (1000 × A_480_ − 1.29 × Chla − 53.78 × Ch1b)/220 (3)

(4) $$\mathrm{Carotenoids}\;\mathrm{extraction}\;\mathrm{efficiency}\;(Y)=\frac{C\times V}{W\times 1000\times 1000}\times 100$$

where Chla represents the concentration of chlorophyll a (µg/mL), Ch1b indicates the concentration of chlorophyll b (µg/mL), C represents the concentration of carotenoids (µg/mL), V stands for the volume of extraction solvent (µL), and W indicates the dry weight of *D. Parva* (mg).

## 3. Results and Discussion

### 3.1. Regression Model and Statistical Test

**Figure 1:**
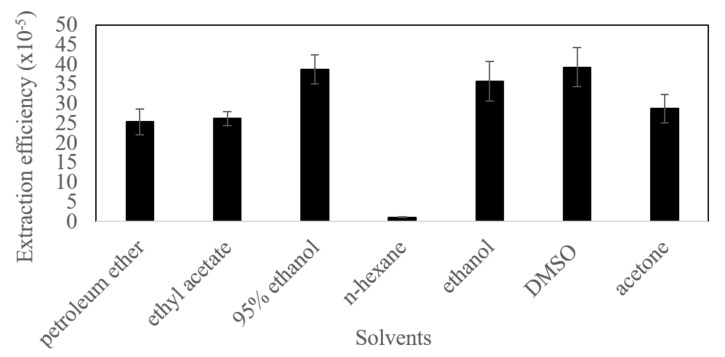
Extraction efficiency of seven commonly used solvents.

The extraction efficiencies of 7 commonly used solvents are shown in Figure 1. The results indicated that DMSO and 95% ethanol had higher extraction efficiencies (0.03926% and 0.03868%). Therefore, these two solvents were selected for further study. The previous studies also reported extracting carotenoids from microalgae with high efficiency using these two solvents. The efficiency of pulsed electric field-assisted extraction combined with DMSO in extracting carotenoids from microalgae *Tetraselmis chui* (Chlorophyta) and *Phaeodactylum tricornutum* (Bacillariophyta) was higher [45]. The cosmeceutical potential of ethanol extract including carotenoids (astaxanthin, β-carotene, canthaxanthin, violaxanthin, zeaxanthin) from microalga *Nannochloropsis* sp. (Eustigmatophyceae) G1-5 isolated from the Republic of Korea was investigated [46]. It was speculated that DMSO and ethanol had higher extraction efficiency due to their highly polar organic solvents.

Using CCD to optimize the carotenoid extraction conditions, 20 group tests were carried out, and the results of the extraction efficiency are depicted in Table 2. The second-order polynomial regression equation showing the connection among carotenoid extraction efficiency (Y) and three variables, time (A), temperature (B) and the proportion of mixed solvent (C), is shown in the following equation:

Y = 34.81 + 0.012A + B + 0.5C − 0.25AB + 0.038AC + 0.15BC − 1.07A^2^ + 1.75B^2^ − 1.34C^2^ − 0.063ABC − 4.89A^2^B + 0.14A^2^C + 0.26AB^2^

The experimental data in Table 2 were analyzed by Design-Expert 10.0.4.0 software to determine the significance. As shown in Table 3, the F-value of the model was 13.31, and the *p*-value was 0.0023 (less than 0.01), which indicated that the selected regression model was extremely significant. In this case, A^2^, B^2^, C^2^ and A^2^B were very significant or significant terms. Meantime, the *p*-value of the lack of fit (0.3064) indicated that the lack of fit was not significantly associated with the pure error. The coefficient R^2^ represents the reliability of the model. The value of R^2^ (0.9665) indicated that the data showed a good agreement for the model, and this model had a huge potential for the prediction of the response value.

**Figure 2:**
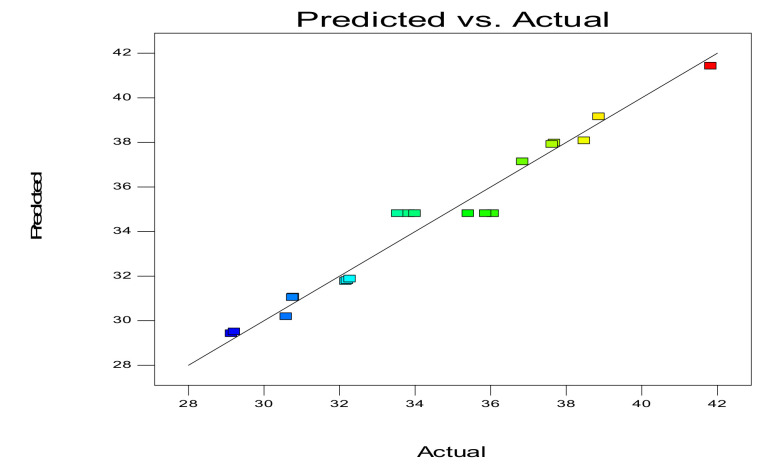
Comparison of predicted and actual response values.

Figure 2 shows the relationship between the theoretical value and the actual response value. The straight line indicates that the theoretical value was roughly equal to the experimental value. As shown in Figure 2, most of the experimental values were distributed in close proximity to a straight line, which suggested that there was little difference between the experimental values and the theoretical values.

The previous studies reported many conventional optimization studies. For enhancing the biomass production of microalga *Mychonastes homosphaera* (formerly *Chlorella minutissima*) (Chlorophyta), BBM and BG-11 were identified as the potential media, and the suitable concentrations of nitrate, phosphate and glycerol were 0.375 g/L, 0.16 g/L and 12.5 g/L by a single factor experiment [47]. The effect of different culture conditions such as light, pH, shaking time and temperature on biomass productivity and growth rate was studied in *Halochlorella rubescens* (formerly *Scenedesmus rubescens*) (Chlorophyta), and the maximum biomass productivity was obtained under the most optimal conditions (white light of 36 W for 16 h, pH 8, 24 h shaking time and 26 °C) by a single factor experiment [48]. With the progress of methodology, many advanced optimization methods such as orthogonal design and response surface methodology (RSM) emerged. An orthogonal design was used to obtain the optimal experimental conditions in iodine adsorption [49]. An orthogonal design was used to optimize light irradiance and the ratio of photoperiods and LEDs in order to increase the photosynthetic capacity and growth of cucumber seedlings by LED illumination [50].

As a more advanced optimization method, RSM is a collection of mathematical and statistical techniques based on the fit of a polynomial equation to the experimental data which describes the behavior of a data set with the objective of making statistical previsions [51]. RSM includes three kinds of designs, Plackett-Burman (PB), CCD and Box-Behnken Design (BBD), which have been widely used for optimization tests. An optimization assay was performed using RSM with CCD for microemulsion-assisted extraction of carotenoids from watermelon pulp, and it was found that the CCD methods could be successfully performed compared to conventional solvent extraction [52]. CCD was successfully used to determine the optimum supplementation of organic carbon and nitrogen in new MSW media [53]. Under optimal medium conditions designed using BBD, a form of RSM, biomass, beta-carotene and lipid yield were increased by 2.17 fold, 1.45 fold and 1.56 fold, respectively [54]. A PB design was used to optimize the factors affecting polyphenol extraction from *Pleioblastus amarus* (Keng) shell, such as ethanol concentration, extraction temperature, liquid to solid ratio, extraction time and reflux extraction times [55].

### 3.2. Interaction among Influence Factors and Confirmation of Optimal Conditions

**Figure 3:**
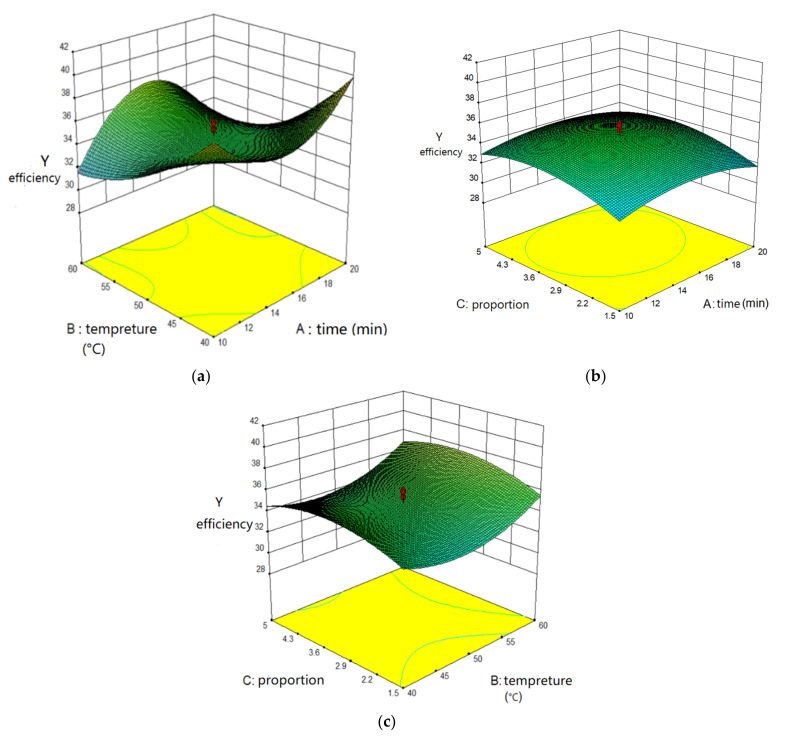
Response using Central Composite Design obtained by plotting: (**a**) time and temperature; (**b**) time and proportions of mixed solvent; (**c**) temperature and proportions of mixed solvent.

Three-dimensional response surfaces were generated to investigate the interaction among three influence factors and to determine the ideal value of each influencing factor for the maximum extraction efficiency of carotenoids. Figure 3 shows the effect of two influencing factors on extraction efficiency.

In Figure 3a, the oval contour lines were dense, and the eccentricity of the flat ellipse was large, which indicated that the influence of time and temperature on extraction efficiency was complex. The pattern of response surface with a large slope was wavy. With the increase of extraction time, the response surface formed a steep slope, which suggested that the interaction of time and temperature had a great influence on extraction efficiency.

In Figure 3b, the contour lines were sparse and approximately oval, and the response surface was spherical with a flat slope. The results showed that time and the proportion of mixed solvent had little effect on extraction efficiency. The cross effect on extraction efficiency was weak.

In Figure 3c, the contour lines were dense and oval, and the response surface was wavy with a flat inclination of the surface. It was shown that two interaction terms (temperature and the proportion of mixed solvent) had little influence on extraction efficiency.

In summary, the effects of three factors (time, temperature and the proportion of mixed solvent) on the extraction efficiency of carotenoids were complex. A^2^, B^2^, C^2^ and A^2^B were very significant or significant terms. In the future, more organic solvents will be investigated regarding their extraction efficiency.

### 3.3. Response Optimization and Validation

Through the analysis of Design-Expert software, the optimal values of the corresponding influencing factors could be obtained for the maximum extraction efficiency of carotenoids from *D. parva*. The optimal conditions were as follows: extraction time of 20 min, temperature of 40 °C, and a proportion of mixed solvent of DMSO: 95% ethanol = 3.64:1. Under the optimal conditions, the theoretical extraction efficiency of carotenoids was 0.0400%. Then, the optimal extraction efficiency under the optimal extraction conditions was subjected to verification. The actual maximum extraction efficiency (0.0464%) was obtained. The actual maximum was essentially in agreement with the theoretical maximum for the extraction efficiency of carotenoids.

### 3.4. Related Research on Carotenoid Extraction from Microalgae

Carotenoids are antioxidant compounds that have been used for many industrial applications. The halophilic microalga *D. parva* is rich in natural carotenoids. A solvent-based extraction method using a solvent mixture of acetone/ethanol/hexane (2/1/1 vol.) and a method using supercritical CO_2_ to extract β-carotene from *D*. *salina* were compared based on environmental and economic perspectives [56]. The results indicated that the potential advantages of the supercritical method (lower energy consumption and greenhouse gas emission) did not balance the disadvantage (low extraction yield) [56]. Monte et al. used n-heptane for the extraction of carotenoids from *D*. *salina* [57]. Rammuni et al. summarized the conventional and modern extraction methods used for the recovery of β-carotene from *D*. *salina* and highlighted the sustainability of integrated co-production of biofuels and carotenoids [58]. However, few studies were reported about the optimization of extraction conditions of carotenoids from *D. parva*, which is rich in oil and carotenoids. Here, the CCD method, a widely used method, was used to optimize the extraction conditions [59,60]. Firstly, the extraction efficiencies of 7 kinds of solvents (petroleum ether, ethyl acetate, 95% ethanol, n-hexane, ethanol, DMSO and acetone) were compared. Secondly, two types of solvents with higher extraction efficiencies (DMSO and 95% ethanol) were mixed as a mixed solvent. The CCD method was used to optimize the levels of three influence factors (time, temperature and the mixed proportions) in order to obtain the best extraction efficiency. The optimized extraction temperature of 40 °C was significantly lower than the standard temperature of 60 °C, which could obviously save energy. The optimized extraction efficiency of 0.0464% improved by 18.19% and 19.96% compared with that of DMSO (0.03926%) and 95% ethanol (0.03868%). In a word, lower energy consumption and higher extraction efficiencies were obtained through CCD optimization.

Many environmental factors could affect the carotenoid content of microalgae. Nitrate concentrations, salinity and light quality could affect carotenoid content in *Dunaliella salina* [61]. High light intensity in combination with nitrogen limitation could result in maximal carotenoid yield [62]. The highest carotenoid yield was obtained under nitrogen and salinity stress conditions in *Auxenochlorella protothecoides* (formerly *Chlorella protothecoides*) (Chlorophyta) [63]. Therefore, the regulation of carotenoid content of microalgae is complex, which accounted for the low extraction efficiency in this study.

## 4. Conclusions

In conclusion, we obtained optimized extraction efficiencies and extraction conditions through the CCD method. This study would be helpful for the extraction of carotenoids from *D. parva* and the application of *D. parva*, which is rich in oil and carotenoids.

## Figures and Tables

**Table 1:** The levels of the variables in this study.

Factors	Levels				
	−α	−1	0	+1	+α
A (time, min)	6.59	10	15	20	23.41
B (temperature, °C)	33.18	40	50	60	66.82
C (mixed proportion of DMSO and 95% ethanol)	0.3:1	1.5:1	3.25:1	5:1	6.3:1

**Table 2:** Experimental results of CCD design.

Group	A (Time, Min)	B (Temperature, °C)	C (Proportion of Mixed Solvent)	Actual Extraction Efficiency (Y, 10^−5^)	Predictive Extraction Efficiency (Y, 10^−5^)
1	10	60	5:1	30.78	31.07
9	20	60	5:1	34.00	34.81
2	15	50	3.25:1	35.87	34.81
3	15	50	3.25:1	38.87	39.16
4	20	40	5:1	32.17	31.77
5	6.59	50	3.25:1	33.54	34.81
6	15	50	3.25:1	36.85	37.14
7	10	40	1.5:1	35.41	34.81
8	15	50	3.25:1	30.76	31.05
10	23.4	50	3.25:1	32.21	31.81
11	10	40	5:1	37.63	37.92
12	10	60	1.5:1	29.14	29.43
13	15	50	6.19:1	32.28	31.88
14	15	66.8	3.25:1	41.83	41.43
15	15	50	3.25:1	33.84	34.81
16	15	33.18	3.25:1	38.48	38.08
17	15	50	0.31:1	30.59	30.19
18	20	60	1.5:1	29.22	29.51
19	15	50	3.25:1	36.07	34.81
20	20	40	1.5:1	37.69	37.98

**Table 3:** Analysis of variance for the model.

Source	Sum of Squares	d. f.	Mean Square	F-Value	*p*-Value	Significance
Model	227.97	13	17.54	13.31	0.0023	**
A	0.0008	1	0.0008	0.0006071	0.9811	
B	5.61	1	5.61	4.26	0.0846	
C	1.43	1	1.43	1.08	0.3380	
AB	0.51	1	0.51	0.39	0.5567	
AC	0.011	1	0.011	0.008537	0.9294	
BC	0.19	1	0.19	0.14	0.7200	
A^2^	16.48	1	16.48	12.51	0.0123	*
B^2^	43.97	1	43.97	33.37	0.0012	**
C^2^	25.73	1	25.73	19.53	0.0045	*
ABC	0.031	1	0.031	0.024	0.8827	
A^2^B	79.19	1	79.19	60.09	0.0002	**
A^2^C	0.065	1	0.065	0.049	0.8316	
B^2^A	0.22	1	0.22	0.16	0.6993	
Residual	7.91	1	1.32	-	-	
Lack of fit	1.63	1	1.63	1.30	0.3064	
Pure error	6.28	1	1.26	-	-	
Sum	235.88	-	-	-	-	
R^2^	0.9665	-	-	-	-	
Adj R^2^	0.8939	-	-	-	-	
Precision	12.496	-	-	-	-	

Note: * (*p* < 0.05) represents significant findings, ** (*p* < 0.01) represents extremely significant findings, - represents no data for this item, and d. f. represents degrees of freedom.

## Data Availability

Not applicable.

## Sample Availability

Samples of the carotenoids are available from the authors.
